# Antibiotic prophylaxis before dental procedures to prevent infective endocarditis: a systematic review

**DOI:** 10.1007/s15010-022-01900-0

**Published:** 2022-08-16

**Authors:** Judith Bergadà-Pijuan, Michelle Frank, Sara Boroumand, Frédérique Hovaguimian, Carlos A. Mestres, Robert Bauernschmitt, Thierry Carrel, Bernd Stadlinger, Frank Ruschitzka, Annelies S. Zinkernagel, Roger D. Kouyos, Barbara Hasse

**Affiliations:** 1 Department of Infectious Diseases and Hospital Epidemiology, University Hospital Zurich, University of Zurich, Raemistrasse 100, 8091 Zurich, Switzerland; 2 Department of Cardiology, University Heart Center, University Hospital Zurich, University of Zurich, Zurich, Switzerland; 3 Clinic for Cardiac Surgery, University Hospital Zurich, University of Zurich, Zurich, Switzerland; 4 Department of Public and Global Health, Epidemiology, Biostatistics and Prevention Institute, University of Zurich, Zurich, Switzerland; 5 Center of Dental Medicine, University of Zurich, Zurich, Switzerland

**Keywords:** Infective endocarditis, Dental procedure, Endocarditis prophylaxis, Endocarditis guidelines, Antibiotic prophylaxis prior dental procedure, High-risk patients

## Abstract

**Purpose:**

Infective endocarditis (IE) is a severe bacterial infection. As a measure of prevention, the administration of antibiotic prophylaxis (AP) prior to dental procedures was recommended in the past. However, between 2007 and 2009, guidelines for IE prophylaxis changed all around the word, limiting or supporting the complete cessation of AP. It remains unclear whether AP is effective or not against IE.

**Methods:**

We conducted a systematic review whether the administration of AP in adults before any dental procedure, compared to the non-administration of such drugs, has an effect on the risk of developing IE. We searched for studies in the Cochrane Central Register of Controlled Trials (CENTRAL), MEDLINE via OVID, and EMBASE. Two different authors filtered articles independently and data extraction was performed based on a pre-defined protocol.

**Results:**

The only cohort study meeting our criteria included patients at high-risk of IE. Analysis of the extracted data showed a non-significant decrease in the risk of IE when high-risk patients take AP prior to invasive dental procedures (RR 0.39, *p*-value 0.11). We did not find other studies including patients at low or moderate risk of IE. Qualitative evaluation of the excluded articles reveals diversity of results and suggests that most of the state-of-the-art articles are underpowered.

**Conclusions:**

Evidence to support or discourage the use of AP prior to dental procedures as a prevention for IE is very low. New high-quality studies are needed, even though such studies would require big settings and might not be immediately feasible.

**Supplementary Information:**

The online version contains supplementary material available at 10.1007/s15010-022-01900-0.

## Introduction

Infective endocarditis (IE) is a severe bacterial infection of the heart valves that often occurs on congenitally malformed or degenerated cardiac valves with or without dysfunction [1]. Due to the high mortality rate of up to 30%, the disease has become a major threat of modern medicine [2, 3]. To prevent IE, the American Heart Association (AHA) suggested the administration of antibiotic prophylaxis (AP) before invasive medical or dental procedures since 1955 [4]. The rationale behind the use of AP prior to dental procedures is that circulating doses of antibiotics would prevent the development of transient bacteremia due to oral streptococci and, therefore, such bacteria would not attach onto the endocardium and cause IE [5, 6]. In a study from 2014 [7], 277 prescriptions of AP were needed to prevent one case of IE. However, the proportion of IE cases arising from dental procedures is arguable, and while some modeling studies consider AP to be cost-effective [8, 9], other studies report that the potential benefits of AP are less than the adverse effects [10]. State-of-the-art analyses worldwide report conflicting results in favor or against the use of AP before dental procedures [11–13]. Nevertheless, these practices were adopted in global agreement and continued for years. Recent concerns about drug adverse reactions and antibiotic resistance led to important modifications of the guidelines. In 2007, the AHA restricted AP to patients at high risk of IE who undergo invasive dental procedures [14]. In 2009, a very similar guideline was published by the European Society of Cardiology (ESC) [15], whereby in 2008, the UK National Institute for Health and Care Excellence (NICE) recommended the complete cessation of AP prior to dental procedures [16]. Although this profound change in clinical practice has been implemented in the 2015 ESC guidelines [17], concerns have been raised regarding the poor quality of the available evidence, which mostly relies on underpowered and methodologically flawed studies [18]. Thus, an extensive systematic review is needed to summarize all the evidence on this question and to assess whether the current restrictions in the use of AP are justified.

## Methods

We used the PRISMA guidelines (http://www.prisma-statement.org/) to identify, select, appraise, and synthesize studies for this systematic review. The study protocol was registered at the Prospective Register of Systematic Reviews (PROSPERO; CRD42020175398). Eligibility criteria, outcomes and statistical methods were pre-defined.

### PICOT—eligibility criteria

Our study population included individuals older than 18 years (adults) that underwent any kind of dental procedure. Animal studies and studies involving children were excluded from our research. As intervention, we considered the administration (e.g., oral or intravenous) of AP (e.g., amoxicillin, vancomycin or other antimicrobial treatments) prior to a dental procedure. The control group included patients that received no drugs or a placebo. The main outcome was definite IE as defined by the Duke criteria [17].

### Information sources and search strategy

We searched the three main bibliographic databases: the Cochrane Central Register of Controlled Trials (CENTRAL), MEDLINE via OVID, and EMBASE. The search strategy included headings, title/abstract keywords and mesh terms related to dental procedures, bacterial endocarditis and AP (see detailed search strategies in Supplementary File 1).

For this review, we considered only randomized clinical trials (RCTs) (if available in the field of research) and prospective cohort studies written in English. Nevertheless, we also looked for additional reports by hand-searching the bibliographies of systematic reviews and meta-analyses within the field of our research topic. We included only studies published after 2000, since there has been an important increase in the diagnostic specificity after 2000 with the use of the revised Duke criteria [17] reducing the number of false IE cases. Moreover, the diagnostic tools (echocardiography, Cardiac CT, PET/CT, microbiology techniques) for IE diagnosis and also the dental practices have improved over years [19, 20]. Furthermore, median population age has increased [21] and criteria/standards required to report clinical trials and meta-analyses have changed [22–24].

### Selection process, data extraction and data items

One of the authors searched the databases to find available studies and excluded those publications which, based on their title or abstract, did not meet our inclusion criteria. This person, in parallel with another author, screened the full text of the remaining publications. Both researchers worked independently, selecting only studies meeting the inclusion criteria. Data were extracted from each specific study by the two researchers working separately. Any disagreement was solved with the help of a third author.

We extracted information about the year of publication, study design, number of participants, inclusion and exclusion criteria for patients, and antimicrobial agents used for dental prophylaxis (if applicable). Finally, we extracted the number of IE cases in intervention and control groups in relation to the patients assigned to each of the groups (Table 1).

**Table 1:** Data extraction for included study

Author, year	PMID	Type of study	Country	Study time period	Population	Number of procedures	Patients with AP and IE	Patients with AP and w/o IE	Patients w/o AP and IE	Patients w/o AP and w/o IE
Tubiana, 2017	28,882,817	Cohort study	France	2008–2014	Adults aged more than 18 years with prosthetic heart valves and undergoing invasive dental procedures	103,463	4	52,276	10	51,173

Tubiana et al*.* study includes adults with prosthetic heart valves who undergo invasive and non-invasive dental procedures. For the analysis, we considered only the invasive dental procedures. The study describes the total number of patients and the total number of dental procedures. It must be noted, however, that a few patients received multiple dental procedures during the study periods. For this reason, we considered the total number of dental procedures, and not solely the number of patients

*AP* antibiotic prophylaxis; *IE* infective endocarditis; *w/o* with or without

We assessed the risk of bias for the included publication by the risk of bias tool for observational studies from Cochrane (Table 2).

**Table 2:** Risk of bias summary of included study

	Tubiana et al., 2017
Definition of cases	Catchment area (France) and time period (2008–2014) clearly specified Cases are the patients classified within the category “invasive dental procedure with antibiotic prophylaxis” Criteria for the classification of patients in each category is clearly stated In addition to the number of patients per category, the study also reports the total number of dental procedures
Definition of controls	Catchment area (France) and time period (2008–2014) clearly specified. These are the same as for the cases Controls are the patients classified within the category: “invasive dental procedure without antibiotic prophylaxis”
Characteristics of each group described?	Yes Adults > 18 years, living in France and with prosthetic heart valves Percentage of males/females, different age groups and medical conditions (e.g., diabetes) were also reported
Groups recruited at common stage, in the same manner?	Yes Cases and controls were recruited from the same nationwide cohort, which included only adults with prosthetic heart valves and without any previous discharge diagnosis for oral streptococcal IE Cases and controls were also recruited during the same time period, based on the administration (or not) of AP on the days before a dental procedure
Sampling strategy	Clearly specified Cases: patients that took antibiotics against oral streptococci in the 21 days prior to the dental procedure Controls: patients that did not take antibiotics against oral streptococci during this time period
Is the group representative of the population of interest?	Yes The cohort of analysis includes adults with prosthetic heart valves that underwent at least one dental procedure
Duration of follow-up	Median follow-up is 1.7 years (interquartile range 0.6–3.2 years) Follow-up was done until end of study (December 2014), or until loss of follow-up, or until one of these endpoints: oral streptococcal IE, death, or hospital admission for valve replacement
Outcome assessment	Outcome: oral streptococcal endocarditis Outcome is defined as the first hospital admission with a primary discharge diagnosis of IE using ICD-10 codes, combined with another secondary discharge diagnosis of streptococcal infection This definition is based on other previous studies The assessment of IE is performed considering the three months after an invasive dental procedure
Overall risk of bias	High

### Definitions

We defined patients at high risk of IE [20, 25].

- if they had undergone a prior prosthetic valve replacement/implantation (including transcatheter aortic valve) or a surgical valve repair or intervention (e.g., Mitraclip).
- if they had a previous episode of IE,
- if they had suffered from any type of cyanotic congenital heart disease (CHD) and/or underwent repair with prosthetic material in the 6 months before or lifelong in case of a residual shunt or valvular regurgitation.

We rated patients with a previous history of rheumatic fever, patients with unrepaired congenital anomalies of the heart valves and patients with bicuspid aortic valves, mitral valve prolapse and calcific aortic stenosis at moderate risk for IE [20]. Other heart conditions were rated at low or unknown risk.

### Data analysis

Since we found only one relevant publication, we provide a data summary using qualitative assessment.

### Assessment of excluded publications

Due to the very low number of studies fulfilling our inclusion criteria, and therefore the current lack of evidence, we considered the excluded publications as a descriptive source of information for the discussion. We provide a summary of these studies in Table 3.

**Table 3:** Qualitative evidence of excluded studies

Author, year	PMID	Type of study	Country	Study time period	Statistical results, 95% CI	Results	Reason for exclusion from quantitative analysis
Oliver, 2004 [48]	15,106,220	Systematic Review and Meta-Analysis	Evidence collected from all around the world	1966–2002	OR 1.62, CI 0.57–4.57	*No* (statistical) *evidence* to support or discourage IE Prophylaxis	Only one study is included; study dates back before year 2000
Agha, 2005 [49]	15,951,458	Decision Model Study (Markov Model)	USA	55 years horizon	Clarithromycin: QALY 0.001125 Cephalexin: QALY 0.000827 Clindamycin: QALY 0.001118 Amoxicillin: QALY -0.00303 Cefazolin: QALY 0.000827 Ampicillin: QALY -0.00303 CI not reported	AP is cost-effective for preventing IE when using clarithromycin, cephalexin or clindamycin Use of amoxicillin and ampicillin for IE prophylaxis is not safe	It is not an RCT, prospective cohort study or a case–control study. It is a decision model study evaluating the cost-effectiveness of AP
Duval, 2006 [35]	16,705,565	Cohort Study	France	1998–1999	RR 0.309, CI 0.02–4.94	AP could *reduce* the risk of IE by 70% in high-risk patients (results are statistically *not significant*)	Patients undergoing dental procedures are not the same as patients with/without IE. Instead, two different cohorts are used. Therefore, it’s not an RCT, prospective cohort study nor case–control study
Lockhart, 2007 [50]	17,403,736	Systematic Review	Evidence collected from all around the world	1966–2005	Not available	*No* (statistical) *evidence* to support or discourage IE prophylaxis	Included studies date back before year 2000, or are descriptive/out of our topic
Schwartz, [51]	17,904,722	Systematic Review Meta-Analysis	Evidence collected from all around the world	1997–2007	Not available	*No* (statistical) *evidence* to support or discourage IE prophylaxis	No studies were found in which outcome was IE; authors assessed bacteremia as an outcome (out of our topic)
Duval, 2008 [52]	18,353,264	Systematic Review	Evidence collected from all around the world	1992–2008	Not available	*No* (statistical) *evidence* to support or discourage IE prophylaxis	Included studies date back before year 2000, or are descriptive/out of our topic
Oliver, 2008 [53]	18,843,649	Systematic Review and Meta-Analysis	Evidence collected from all around the world	1950–2008	OR 1.62, CI 0.57–4.57	*No* (statistical) *evidence* to support or discourage IE prophylaxis	Only one study is included; study dates back before year 2000
Ellervall, 2010 [54]	20,134,479	Systematic Review and Meta-Analysis	Evidence collected from all around the world	1996–2009	First time IE episode OR 0.51 (95% CI 0.11 2.29) Recurrent IE episodes OR 2.13 (95% CI 0.48–9.44)	*No* (statistical) *evidence* to support or discourage IE prophylaxis	Only one study is included; study dates back before year 2000
Thornhill, 2011 [55]	21,540,258	Population-based Study (Temporal Trend Study)	UK	2004–2010	Difference in annual percentage change of IE cases before and after change of guidelines: 1.1 (95% CI -3.9–1.9)	*No increase* in cases of IE since restriction of AP in NICE guidelines (2008)	It is not an RCT, prospective cohort study nor case–control study. It is a time-trend analysis
Desimone, 2012 [56]	22,689,929	Population-based Study (Temporal Trend Study)	USA	1999–2010	1999–2002: IR 3.19, (95% CI 1.20–5.17) 2003–2006: IR 2.48, (95% CI 0.85–4.10) 2007–2010: IR 0.77, (95% CI 0.00–1.64)	*No increase* in incidence of IE since restriction of AP in AHA guidelines (2007)	It is not an RCT, prospective cohort study nor case–control study. It is a time-trend analysis
Glenny, 2013 [1]	24,108,511	Systematic Review and Meta-Analysis	Evidence collected from all around the world	1946–2013	OR 1.62, CI 0.57–4.57	*No* (statistical) *evidence* to support or discourage IE prophylaxis	Only one study is included; study dates back before year 2000
Dayer, 2015 [7]	25,467,569	Population-based Study (Temporal Trend Study)	UK	2000–2013	Increase in incidence of IE: 0.11 cases per 10 Mio people per month (95% CI 0.05–0.16)	*Increase* in incidence of IE since restriction of AP in NICE guidelines (2008) The increase is seen for individuals at high risk and low risk of IE	It is not an RCT, prospective cohort study nor case–control study. It is a retrospective secular trend study
Chen, 2015 [12]	26,512,586	Cohort Study (Case-crossover)	Taiwan	1999–2012	Tooth extraction OR 0.56, (95% CI 0.22–1.41) Dental scaling: OR 0.85, (95% CI 0.54–1.35 Periodontal: OR 1.24, (95% CI 0.59–2.62 Endodontic: OR 1.20, (95% CI 0.64–2.25	Dental procedures do not contribute to the risk of IE Results are *against using AP* for dental procedures	Cases and Controls are the same patients, but in different time periods. It is a retrospective study
Chirillo, 2016 [57]	27,595,678	Cohort Study	Italy	2007–2010	Not available	*No* (statistical) *evidence* to support or discourage IE prophylaxis	No control group is provided. Therefore, data cannot be extracted and main outcome of the study is not IE
Franklin, 2016 [58]	27,840,334	Decision Model Study	UK	50-year horizon	Amoxicillin: QALY 0.0012 (95% CI 0.000–0.003) Clindamycin: QALY 0.0010 (95% CI 0.000–0.002)	Prophylaxis is cost-effective for preventing IE	It is not an RCT, prospective cohort study nor case–control study. It is a decision model study evaluating the cost-effectiveness of AP
Cahill, 2017 [18]	28,213,367	Systematic Review and Meta-Analysis	Evidence collected from all around the world	1945–2016	Observational studies for IE: OR 0.59 (95% CI 0.27–1.30)	The evidence for the use of AP is *unclear* There is a limited protective effect of AP for IE (statistically *not significant*), but AP is effective (statistically) in reducing the bacteremia	Included studies are time-trend analyses (excluded according to our criteria), trials that assess bacteremia instead of IE as an outcome (out of our topic), and observational studies published before 2000
Thornhill, 2018 [13]	30,409,564	Population-based Study (Temporal Trend Study)	USA	2003–2015	Increase in incidence of IE: High-risk: 177% CI 66%–361% Moderate risk: 75% (95% CI 3%–200%) Low risk: 12% (95% CI -29%–76%)	The change in AHA Guidelines is related with a *significant increase* in the incidence of IE for patients at *high-risk* and *moderate-risk* The increase is not significant for patients at low risk of IE	It is not an RCT, prospective cohort study nor case–control study. It is a time-trend study that uses a Poisson model analysis
Karacaglar, 2019 [44]	31,464,231	Cohort Study (retrospective)	Turkey	2016–2018	Not available	*No* (statistical) *evidence* to support or discourage IE prophylaxis	Group with IE is not provided. Therefore, data cannot be extracted and main outcome of the study is not IE It is a retrospective study
Quan, 2020 [47]	32,238,164	Population-based Study (Temporal Trend Study)	UK	1998–2017	Increase in incidence of IE:from 22–41 cases per 1 Mio people in 1998 to 42–68 cases per 1 Mio people in 2017	*Increase* in incidence of IE, but this increase does not directly follow the update of the NICE guidelines (2008)	It is not an RCT, prospective cohort study nor case–control study. It is a time-trend study that uses a range of regression models for analysis
Vähäsarja, 2020 [46]	33,014,311	Population-based Study (Temporal Trend Study)	Sweden	2008–2017	Before 2012: 0.344 cases per 10 M people per month CI 0.19–0.50 After 2012: 0.266 cases per 10 M people per month (95% CI 0.12–0.42) Decrease in the slope of the trendline: − 0.08 cases per 10 M people per month (95% CI − 0.30–0.14)	*No increase* in the incidence of IE as a result of the recommendations published in 2012 for the cessation of AP in dentistry	It is not an RCT, prospective cohort study nor case–control study. It is an Interrupted Time Series Analysis (ITSA)
Thornhill, 2020 [45]	33,121,605	Cohort Study	USA	2000–2015	Not available	*No* (statistical) *evidence* to support or discourage IE prophylaxis	Group with IE is not provided. Therefore, data cannot be extracted and main outcome of the study is not IE
Rutherford., 2022 [46]	35,536,541	Systematic Review and Meta-Analysis	Evidence collected from all around the world	1946–2021	OR 1.62, (95% CI 0.57–4.57)	No (statistical) *evidence* to support or discourage IE prophylaxis	Only one study is included; study dates back before year 2000

*AP* antibiotic prophylaxis; *OR* odds ratio; *RR* risk ratio; *HRR* hazard rate ratio; *IR* incidence rate per 100,000 persons-year; *CI* 95% confidence intervals; *QALY* stands for the increase in quality-adjusted life-years per patient

^*^90% CI is reported for this study instead of 95% CI

## Results

**Fig. 1:**
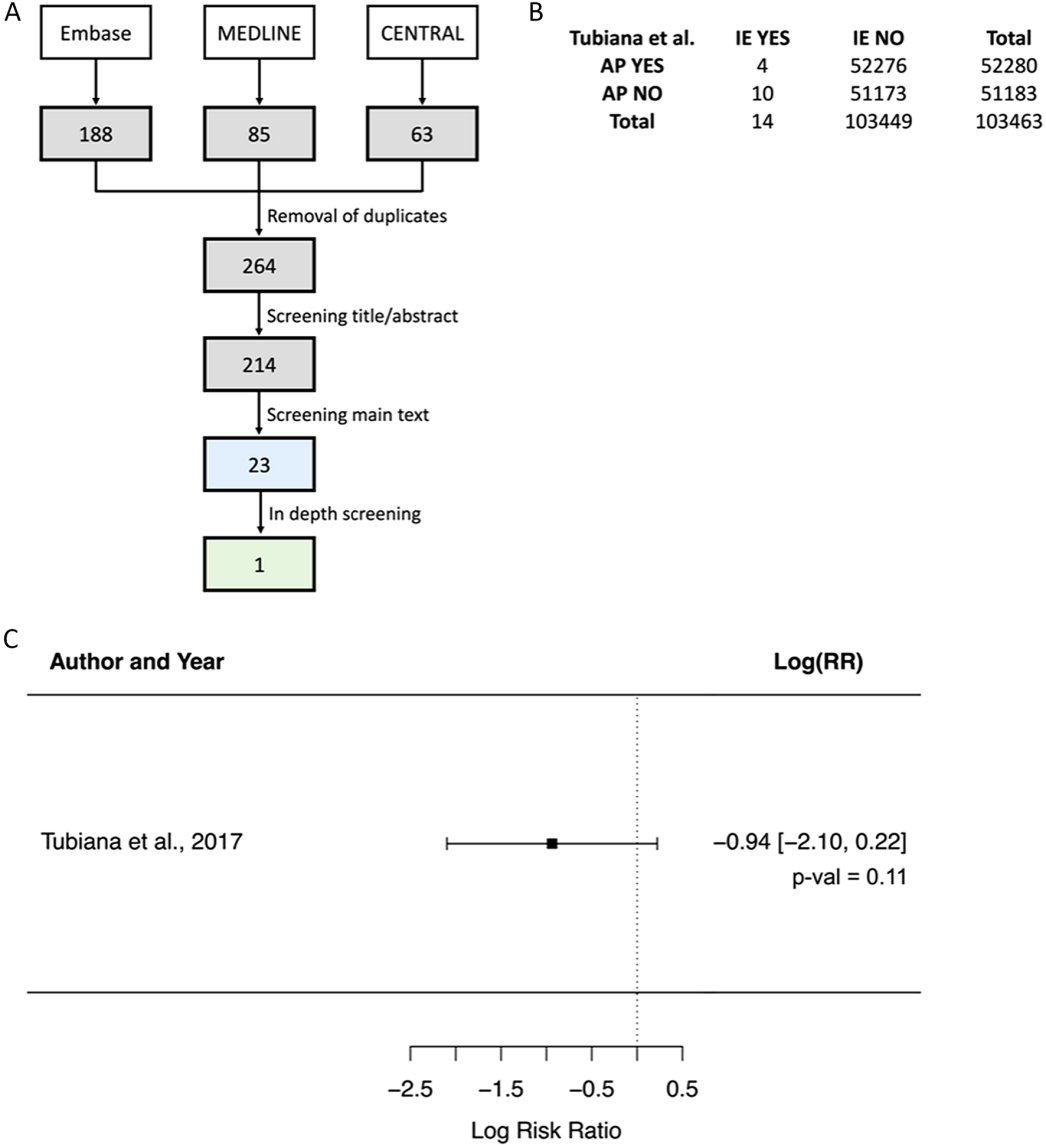
**A** Literature search and filtering process. Numbers correspond to studies under consideration at each step. Green shows the final number of publications that met our inclusion criteria. Blue shows the number of publications for which we clearly describe the reason of inclusion/exclusion. **B** Contingency table for the included publication. **C** Plot of the effects of AP in the risk ratio of developing IE based on one publication

The database search resulted in 63 studies from CENTRAL, 85 studies from MEDLINE and 188 studies from EMBASE (Fig. 1A). After exclusion of duplicates, 264 publications were further assessed. Title and abstract screening resulted in 214 relevant studies, of which 191 were descriptive, provided only qualitative results, or were not considered otherwise eligible (e.g., main outcome was bacteremia, control group was not placebo, etc.). Thus, we found 23 publications that could potentially be included in our research. After assessing each of them individually, only one study met our inclusion criteria.

The only publication fulfilling the inclusion criteria is a prospective cohort study (Table 1) [26]. The observational study has a high risk of bias, as shown in Table 2. *Tubiana *et al. includes adults with prosthetic heart valves who underwent invasive and non-invasive dental procedures. For the present analysis, we considered only the invasive dental procedures. Data extraction and analysis (Fig. 1B, C) show a decrease in the risk of developing IE when high-risk patients received antibiotics prior to an invasive dental procedure, in accordance with the current AHA and ESC Guidelines. Nonetheless, results were not statistically significant. Based on the calculations, taking AP could slightly reduce the overall risk of developing IE in high-risk patients (*p*-value 0.11; RR 0.39). Overall, these results provided only a very weak evidence of an effect of AP on the risk of developing IE in high-risk patients. In this study, all patients had prosthetic cardiac valves and hence were at high risk of developing infective endocarditis. Patients at low and moderate risk of IE were not included and therefore we are unable to assess the effects of AP prior to dental procedures in these groups of patients.

## Discussion

The present investigation suggests that prescribing AP before dental procedures may prevent the risk of developing IE in high-risk patients, based on a single prospective cohort study [26]. Therefore, these results are consistent with the current AHA and ESC Guidelines, advising AP in patients at elevated risk of IE who have to undergo a dental procedure [14, 20, 27, 28]. However, no prospective, randomized, placebo-controlled trial has been performed to confirm or refute the usefulness of AP for patients undergoing dental procedures [28]. Evidence is based on observational studies only with a potential risk of bias.

Furthermore, we found no study able to answer this question in patients at moderate or low risk of IE. Hence, it remains unclear whether these patients may benefit from AP. Some case–control studies on the topic are very old and their validity is questionable [29–32]. Literature on the effect of AP in preventing IE in moderate and low-risk patients is scarce. The 2007 AHA Guidelines limited AP to high-risk patients and interventions, especially in the oral and dental area. A time-trend study based on the US population [13] suggested that following the change of the AHA guidelines, incidence of IE did not change in the low-risk population, but it showed a modest yet statistically significant increase in the moderate-risk population, and a dramatic increase in the high-risk population. The study did not show a causal relationship between the fall in AP prescription and the increase in IE incidence. However, it provided support to the 2007 AHA Guidelines while a revision of criteria for the classification of moderate-risk patients was recommended. Similarly, another study based on the UK population [33] suggested the need of re-evaluating IE risk classification in patients with cardiac conditions, and showed that risk of IE in some moderate-risk individuals was similar to that of high-risk individuals. However, microorganism specific data are lacking in this study rendering interpretation of data difficult.

In 2015, a time-trend study in the UK [7] found a highly significant fall in AP prescription and a significant increase in the incidence of IE following the implementation of the NICE guidelines [16]. By contrast, a case-crossover design based on the Taiwanese population [12] showed that the association between the risk of IE and dental procedures was not statistically significant, even after adjusting for antibiotic use. The publication argued against the use of AP for dental procedures, claiming that dental procedures do not significantly contribute to the risk of IE. A similar case-crossover design based in Israel [34] came to the same conclusion. In 2006, a study of the French population [35] found a positive effect of AP for at-risk dental procedures in patients with predisposing cardiac conditions. The same study, nevertheless, argued that a high number of patients would need AP to avoid one single case of IE [35]. The most recent evidence in this field is from a Swedish nationwide cohort study. The study did not find an increased incidence of oral streptococcal endocarditis among high-risk individuals after promoting the cessation of AP in dentistry for the prevention of infective endocarditis among high-risk individuals [36]. However, the registry-based study is questionable since a revision of the recommendations for AP in Swedish dentistry was issued, while the study was ongoing stating that AP could be considered if recommended by the patient’s dentist. Moreover, the information on dental procedures among individual study participants was lacking [36].

Other descriptive reviews reveal that dental procedures cause a minor number of IE, suggesting that AP could only prevent a very low proportion of cases [37–39]. However, estimates regarding the percentage of IE caused by dental procedures are very diverse [8], with some reports claiming a risk of up to 30% in children [40]. Several case–control studies from the 1990s also reported no association between dental procedures and IE. In 1998, a case–control study performed in Philadelphia [29] proved that other factors related to cardiac valve pathologies than dental treatments might contribute to the development of IE. In this study [29], only very few participants received AP and the sample size was too small; thus, the effect of AP in the risk of developing IE was not conclusive. In 1995, a case–control study performed in France [30] stated that dental procedures were overall not related to an increased risk of IE, even though specific treatments such as scaling and root canal displayed trends towards a more elevated IE risk. In this study, however, authors did not consider those patients with IE who died, possibly leading to a biased analysis [1]. In 1992, another case–control study in the Netherlands [31] provided no evidence supporting that AP prior to an invasive dental procedure in high-risk patients is effective against IE (results were not statistically significant). Similar to the cohort study included in our review, this study only included individuals with known cardiac risks. Opposed to these results, another case–control study from 1990, which included only high-risk patients with cardiac lesions [32], reported that the use of AP provided a statistically significant protective effect against IE. In this study, patients with IE who died were also excluded [1].

According to the 2015 ESC Guidelines [20], the rationale behind the prescription of AP was developed in an attempt to prevent the attachment of bacteria to the endocardium during transient bacteremia due to invasive dental procedures. In line, multiple articles have reported an increase of bacteremia after dental procedures and a subsequent decrease when antibiotics are used [41–43]. Furthermore, an extensive meta-analysis published in 2017 [3] showed the results of 21 studies and revealed that AP was associated with a much lower risk ratio for bacteremia as compared to placebo, with highly significant results. Despite these facts, however, a direct causal relationship between dental procedures and IE itself has never been established [44–47].

Taken together, our systematic review indicates a lack of evidence whether AP before dental procedures indeed prevents IE, especially for patients at low and moderate risk. So far, guidelines for the prevention of IE are based on expert opinion [14, 27, 28]. Nonetheless, due to the absence of RCTs and the limited number of conclusive observational studies, the evidence in favor or against the use of AP is scarce. Furthermore, the low incidence of IE [19] makes it difficult to properly investigate the topic, since a high number of patients should be included in the analyses to ensure a sufficient statistical power. In addition, dentists' opinions on this subject differ greatly. Hence, to provide a reliable fundament for future upgrades and improvements of the guidelines [14, 27, 28], it is crucial to perform well-designed and -powered studies that are capable to overcome all limitations mentioned throughout the present systematic review.

## Supplementary Information

Below is the link to the electronic supplementary material.

Supplementary file1 (DOCX 37 KB)

Supplementary file2 (PPTX 176 KB)

## References

[1] Glenny AM, Oliver R, Roberts GJ, Hooper L, Worthington HV (2013). Antibiotics for the prophylaxis of bacterial endocarditis in dentistry. Cochrane Database Syst Rev.

[2] Fernandez Guerrero ML, Gonzalez Lopez JJ, Goyenechea A, Fraile J, de Gorgolas M (2009). Endocarditis caused by *Staphylococcus aureus*: a reappraisal of the epidemiologic, clinical, and pathologic manifestations with analysis of factors determining outcome. Medicine (Baltimore).

[3] Cahill TJ, Prendergast BD (2016). Infective endocarditis. Lancet.

[4] Signs C (1955). PREVENTION of rheumatic fever and bacterial endocarditis through control of streptococcal infections. Pediatrics.

[5] Dajani AS (1998). Prevention of bacterial endocarditis: highlights of the latest recommendations by the American Heart Association. Pediatr Infect Dis J.

[6] Danchin N, Duval X, Leport C (2005). Prophylaxis of infective endocarditis: French recommendations 2002. Heart.

[7] Dayer MJ, Jones S, Prendergast B, Baddour LM, Lockhart PB, Thornhill MH (2015). Incidence of infective endocarditis in England, 2000–13: a secular trend, interrupted time-series analysis. Lancet.

[8] Franklin CD (1992). The aetiology, epidemiology, pathogenesis and changing pattern of infective endocarditis, with a note on prophylaxis. Br Dent J.

[9] Devereux RB, Frary CJ, Kramer-Fox R, Roberts RB, Ruchlin HS (1994). Cost-effectiveness of infective endocarditis prophylaxis for mitral valve prolapse with or without a mitral regurgitant murmur. Am J Cardiol.

[10] Bor DH, Himmelstein DU (1984). Endocarditis prophylaxis for patients with mitral valve prolapse. A quantitative analysis. Am J Med.

[11] Bolger AF (2009). The rationale for the new infective endocarditis guidelines. Curr Cardiol Rep.

[12] Chen PC, Tung YC, Wu PW, Wu LS, Lin YS, Chang CJ (2015). Dental procedures and the risk of infective endocarditis. Medicine (Baltimore).

[13] Thornhill MH, Gibson TB, Cutler E, Dayer MJ, Chu VH, Lockhart PB (2018). Antibiotic prophylaxis and incidence of endocarditis before and after the 2007 AHA recommendations. J Am Coll Cardiol.

[14] Wilson W, Taubert KA, Gewitz M, Lockhart PB, Baddour LM, Levison M, et al. Prevention of infective endocarditis: guidelines from the American Heart Association: a guideline from the American Heart Association Rheumatic Fever, Endocarditis and Kawasaki Disease Committee, Council on Cardiovascular Disease in the Young, and the Council on Clinical Cardiology, Council on Cardiovascular Surgery and Anesthesia, and the Quality of Care and Outcomes Research Interdisciplinary Working Group. J Am Dent Assoc 2007;138(6):739–45, 47–60. 10.14219/jada.archive.2007.0262 10.14219/jada.archive.2007.0262 17545263

[15] Habib G, Hoen B, Tornos P, Thuny F, Prendergast B, Vilacosta I, et al. Guidelines on the prevention, diagnosis, and treatment of infective endocarditis (new version 2009): the Task Force on the Prevention, Diagnosis, and Treatment of Infective Endocarditis of the European Society of Cardiology (ESC). Endorsed by the European Society of Clinical Microbiology and Infectious Diseases (ESCMID) and the International Society of Chemotherapy (ISC) for Infection and Cancer. Eur Heart J. 2009;30(19):2369–413. 10.1093/eurheartj/ehp285 10.1093/eurheartj/ehp285 19713420

[16] (NICE) NIfHaE. Prophylaxis against infective endocarditis: antimicrobial prophylaxis against infective endocarditis in adults and children undergoing interventional procedures. National Institute for Health and Clinical Excellence: Guidance. London; 2008. 21656971

[17] Li JS, Sexton DJ, Mick N, Nettles R, Fowler VG, Ryan T (2000). Proposed modifications to the Duke criteria for the diagnosis of infective endocarditis. Clin Infect Dis.

[18] Cahill TJ, Harrison JL, Jewell P, Onakpoya I, Chambers JB, Dayer M (2017). Antibiotic prophylaxis for infective endocarditis: a systematic review and meta-analysis. Heart.

[19] Baluta MM, Benea EO, Stanescu CM, Vintila MM. Endocarditis in the 21(st) Century. Maedica (Bucur). 2011;6(4):290–7. PMC3391947. PMC3391947 22879844

[20] Habib G, Lancellotti P, Antunes MJ, Bongiorni MG, Casalta JP, Del Zotti F (2015). 2015 ESC Guidelines for the management of infective endocarditis: The Task Force for the Management of Infective Endocarditis of the European Society of Cardiology (ESC). Endorsed by: European Association for Cardio-Thoracic Surgery (EACTS), the European Association of Nuclear Medicine (EANM). Eur Heart J.

[21] Sun YP, O'Gara PT (2018). Cardiovascular conditions predisposing to infective endocarditis: time to reconsider the current risk classification system?. Eur Heart J.

[22] Schulz KF, Altman DG, Moher D (2010). CONSORT 2010 statement: updated guidelines for reporting parallel group randomised trials. J Pharmacol Pharmacother.

[23] Moher D, Cook DJ, Eastwood S, Olkin I, Rennie D, Stroup DF (2000). Improving the quality of reports of meta-analyses of randomised controlled trials: the QUOROM statement. QUOROM group. Br J Surg.

[24] Skrivankova VW, Richmond RC, Woolf BAR, Davies NM, Swanson SA, VanderWeele TJ (2021). Strengthening the reporting of observational studies in epidemiology using mendelian randomisation (STROBE-MR): explanation and elaboration. BMJ.

[25] Ostergaard L, Valeur N, Ihlemann N, Bundgaard H, Gislason G, Torp-Pedersen C (2018). Incidence of infective endocarditis among patients considered at high risk. Eur Heart J.

[26] Tubiana S, Blotiere PO, Hoen B, Lesclous P, Millot S, Rudant J (2017). Dental procedures, antibiotic prophylaxis, and endocarditis among people with prosthetic heart valves: nationwide population based cohort and a case crossover study. BMJ.

[27] Wilson WR, Gewitz M, Lockhart PB, Bolger AF, DeSimone DC, Kazi DS (2021). Prevention of Viridans group streptococcal infective endocarditis: a scientific statement from the American Heart Association. Circulation.

[28] Wilson WR, Gewitz M, Lockhart PB, Bolger AF, DeSimone DC, Kazi DS (2021). Adapted from: Prevention of Viridans group streptococcal infective endocarditis: a scientific statement from the American Heart Association. J Am Dent Assoc.

[29] Strom BL, Abrutyn E, Berlin JA, Kinman JL, Feldman RS, Stolley PD (1998). Dental and cardiac risk factors for infective endocarditis. A population-based, case–control study. Ann Intern Med.

[30] Lacassin F, Hoen B, Leport C, Selton-Suty C, Delahaye F, Goulet V (1995). Procedures associated with infective endocarditis in adults. A case–control study. Eur Heart J.

[31] Van der Meer JT, Van Wijk W, Thompson J, Vandenbroucke JP, Valkenburg HA, Michel MF (1992). Efficacy of antibiotic prophylaxis for prevention of native-valve endocarditis. Lancet.

[32] Imperiale TF, Horwitz RI (1990). Does prophylaxis prevent postdental infective endocarditis? A controlled evaluation of protective efficacy. Am J Med.

[33] Thornhill MH, Jones S, Prendergast B, Baddour LM, Chambers JB, Lockhart PB (2018). Quantifying infective endocarditis risk in patients with predisposing cardiac conditions. Eur Heart J.

[34] Porat Ben-Amy D, Littner M, Siegman-Igra Y (2009). Are dental procedures an important risk factor for infective endocarditis? A case-crossover study. Eur J Clin Microbiol Infect Dis.

[35] Duval X, Alla F, Hoen B, Danielou F, Larrieu S, Delahaye F (2006). Estimated risk of endocarditis in adults with predisposing cardiac conditions undergoing dental procedures with or without antibiotic prophylaxis. Clin Infect Dis.

[36] Vahasarja N, Lund B, Ternhag A, Gotrick B, Olaison L, Hultin M (2022). Infective endocarditis among high-risk individuals - before and after the cessation of antibiotic prophylaxis in dentistry: a national cohort study. Clin Infect Dis.

[37] Pallasch TJ (2003). Antibiotic prophylaxis: problems in paradise. Dent Clin North Am.

[38] Roberts GJ (1999). Dentists are innocent! "Everyday" bacteremia is the real culprit: a review and assessment of the evidence that dental surgical procedures are a principal cause of bacterial endocarditis in children. Pediatr Cardiol.

[39] Guntheroth WG (1984). How important are dental procedures as a cause of infective endocarditis?. Am J Cardiol.

[40] Droz D, Koch L, Lenain A, Michalski H (1997). Bacterial endocarditis: results of a survey in a children's hospital in France. Br Dent J.

[41] Okell CCE, Elliot SD (1935). Bacteriæmia and oral sepsis with special reference to the ætiology of subacute endocarditis. Lancet.

[42] Limeres Posse J, Alvarez Fernandez M, Fernandez Feijoo J, Medina Henriquez J, Lockhart PB, Chu VH (2016). Intravenous amoxicillin/clavulanate for the prevention of bacteraemia following dental procedures: a randomized clinical trial. J Antimicrob Chemother.

[43] Lockhart PB, Brennan MT, Sasser HC, Fox PC, Paster BJ, Bahrani-Mougeot FK (2008). Bacteremia associated with toothbrushing and dental extraction. Circulation.

[44] Karacaglar E, Akgun A, Ciftci O, Altiparmak N, Muderrisoglu H, Haberal M (2019). Adequacy of infective endocarditis prophylaxis before dental procedures among solid organ transplant recipients. Saudi J Kidney Dis Transpl.

[45] Thornhill MH, Gibson TB, Durkin MJ, Dayer MJ, Lockhart PB, O'Gara PT (2020). Prescribing of antibiotic prophylaxis to prevent infective endocarditis. J Am Dent Assoc.

[46] Rutherford SJ, Glenny AM, Roberts G, Hooper L, Worthington HV (2022). Antibiotic prophylaxis for preventing bacterial endocarditis following dental procedures. Cochrane Database Syst Rev.

[47] Quan TP, Muller-Pebody B, Fawcett N, Young BC, Minaji M, Sandoe J (2020). Investigation of the impact of the NICE guidelines regarding antibiotic prophylaxis during invasive dental procedures on the incidence of infective endocarditis in England: an electronic health records study. BMC Med.

[48] Oliver R, Roberts GJ, Hooper L (2004). Penicillins for the prophylaxis of bacterial endocarditis in dentistry. Cochrane Database Syst Rev.

[49] Agha Z, Lofgren RP, VanRuiswyk JV (2005). Is antibiotic prophylaxis for bacterial endocarditis cost-effective?. Med Decis Making.

[50] Lockhart PB, Loven B, Brennan MT, Fox PC. The evidence base for the efficacy of antibiotic prophylaxis in dental practice. J Am Dent Assoc. 2007;138(4):458–74; quiz 534–5, 437. 10.14219/jada.archive.2007.0198 10.14219/jada.archive.2007.0198 17403736

[51] Schwartz AB, Larson EL (2007). Antibiotic prophylaxis and postoperative complications after tooth extraction and implant placement: a review of the literature. J Dent.

[52] Duval X, Leport C (2008). Prophylaxis of infective endocarditis: current tendencies, continuing controversies. Lancet Infect Dis.

[53] Oliver R, Roberts GJ, Hooper L, Worthington HV (2008). Antibiotics for the prophylaxis of bacterial endocarditis in dentistry. Cochrane Database Syst Rev.

[54] Ellervall E, Vinge E, Rohlin M, Knutsson K. Antibiotic prophylaxis in oral healthcare—the agreement between Swedish recommendations and evidence. Br Dent J. 2010;208(3):E5; discussion 114–5. 10.1038/sj.bdj.2010.107 10.1038/sj.bdj.2010.107 20134479

[55] Thornhill MH, Dayer MJ, Forde JM, Corey GR, Chu VH, Couper DJ (2011). Impact of the NICE guideline recommending cessation of antibiotic prophylaxis for prevention of infective endocarditis: before and after study. BMJ.

[56] Desimone DC, Tleyjeh IM, Correa de Sa DD, Anavekar NS, Lahr BD, Sohail MR (2012). Incidence of infective endocarditis caused by viridans group streptococci before and after publication of the 2007 American Heart Association's endocarditis prevention guidelines. Circulation.

[57] Chirillo F, Faggiano P, Cecconi M, Moreo A, Squeri A, Gaddi O (2016). Predisposing cardiac conditions, interventional procedures, and antibiotic prophylaxis among patients with infective endocarditis. Am Heart J.

[58] Franklin M, Wailoo A, Dayer MJ, Jones S, Prendergast B, Baddour LM (2016). The cost-effectiveness of antibiotic prophylaxis for patients at risk of infective endocarditis. Circulation.

