# Supplementary material for: Antibiotic prophylaxis before dental procedures to prevent infective endocarditis: a systematic review
Source: Infection. 2022 Aug 16;51(1):47–59. doi: 10.1007/s15010-022-01900-0 (PMC9879842; doi:10.1007/s15010-022-01900-0)
Supplement: Supplementary file 1 — Supplementary file1 (DOCX 37 KB) [file 15010_2022_1900_MOESM1_ESM.docx]

**SUPPLEMENTARY FILE 1: SEARCH STRATEGY**

**Database: Ovid MEDLINE®**

Search date: May 11, 2022

Search Strategy:

#1 exp Dentistry/ (423977)

#2 (dental procedur* or oral procedur* or maxillofacial procedur* or maxillofacial

surgery or operative dentistry or dental care or oral surgery or dental surgery).tw. (31814)

#3 1 or 2 (436433)

#4 exp Antibiotic Prophylaxis/ (15100)

#5 exp Pre-Exposure Prophylaxis/ (3968)

#6 (antibiotic prophylaxis or prophylaxis or prevention or antibiotic premedic* or

antimicrobial or antibiotic*).tw. (1177120)

#7 4 or 5 or 6 (1181562)

#8 3 and 7 (17501)

#9 exp clinical trials as topic/ or intervention studies/ (374236)

#10 Random Allocation/ (106855)

#11 random*.tw. (1316343)

#12 placebo.tw. (234511)

#13 randomi#ed controlled trial*.pt. (572261)

#14 trial.tw. (697508)

#15 groups.tw. (2378909)

#16 9 or 10 or 11 or 12 or 13 or 14 or 15 (3900594)

#17 exp animals/ not humans.sh. (5007439)

#18 16 not 17 (3363101)

#19 8 and 18 (4187)

#20 exp Endocarditis/ (30052)

#21 (endocarditis or endocarditides).tw. (37163)

#22 (endocardium adj5 (inflamm* or infect*)).tw. (121)

#23 20 or 21 or 22 (43053)

#24 19 and 23 (127)

#25 limit 24 to yr="2000 -Current" (85)

**Database: Embase®**

Search date: May 11, 2022

Search strategy:

#1 ’ dentistry’/exp (141,223)

#2 ’dental procedur*’ OR ‘oral procedur*’ OR ‘maxillofacial procedur*’ OR ‘maxillofacial surgery’ OR ‘operative dentistry’ OR ‘dental care’ OR ‘oral surgery’ OR ‘dental surgery’:ab,ti (262,052)

#3 #1 OR #2 (376,377)

#4 ’prophylaxis’/exp (1,123,081)

#5 ’antibiotic prophylaxis’ OR ‘prophylaxis’ OR ‘prevention’ OR ‘antibiotic premedic*’ OR ‘antimicrobial’ OR ‘antibiotic*’:ab,ti (3,150,648)

#6 #4 OR #5 (3,732,516)

#7 'clinical trial (topic)'/exp OR 'clinical trial'/exp OR random*:ab,ti OR control*:ab,ti OR placebo:ab,ti OR group*:ab,ti OR trial:ab,ti (11,167,059)

#8 ’human’/exp (24,722,899)

#9 ’endocarditis’/exp (57,712)

#10 ’endocarditis’ OR ‘endocarditides’:ab,ti (65,395)

#11 (endocardium NEAR/ (inflamm* OR infect*)):ab,ti (171)

#12 #9 OR #10 OR #11 (65,449)

#13 #3 AND #6 AND #7 AND #8 AND #12 AND [embase]/lim AND [2000-2021]/py (188)

**Database: CENTRAL®**

Search date: May 11, 2022

Search Strategy:

#1 MeSH descriptor: [Dentistry] explode all trees (18883)

#2 (dental procedur* or oral procedur* or maxillofacial procedur* or maxillofacial surgery or operative dentistry or dental care or oral surgery or dental surgery):ti,ab,kw (60885)

#3 #1 or #2 (72473)

#4 MeSH descriptor: [Antibiotic Prophylaxis] explode all trees (1339)

#5 MeSH descriptor: [Pre-Exposure Prophylaxis] explode all trees (245)

#6 (antibiotic prophylaxis or prophylaxis or prevention or antibiotic premedic* or antimicrobial or antibiotic*):ti,ab,kw (232809)

#7 (penicillin or phenoxymethylpenicillin or amoxicillin or aminopenicillin or ampicillin or cefaclor or cephalosporine or cefazolin or ceftriaxone or cephalexin or clindamycin or azithromycin or clarithromycin or roxithromycin or erythromycin or macrolide or vancomycin or metronidazole or nitroimidazole or tetracycline or doxycycline):ti,ab,kw (26804)

#8 #4 or #5 or #6 or #7 (246635)

#9 #3 and #8 (16895)

#10 MeSH descriptor: [Endocarditis] explode all trees (168)

#11 (endocarditis or endocarditides or endocardium):ti,ab,kw (894)

#12 #10 or #11 (894)

#13 #9 and #12 (76)

#14 #13 with Cochrane Library publication date Between Jan 2000 and May 2022 (63)

**Notes**

Numbers within parenthesis denote the total number of publications found for each search.
