# Supplementary material for: Antibiotic prophylaxis before dental procedures to prevent infective endocarditis: a systematic review
Source: Infection. 2022 Aug 16;51(1):47–59. doi: 10.1007/s15010-022-01900-0 (PMC9879842; doi:10.1007/s15010-022-01900-0)
Supplement: Supplementary file 2 — Supplementary file2 (PPTX 176 KB) [file 15010_2022_1900_MOESM2_ESM.pptx]

## Slide 1
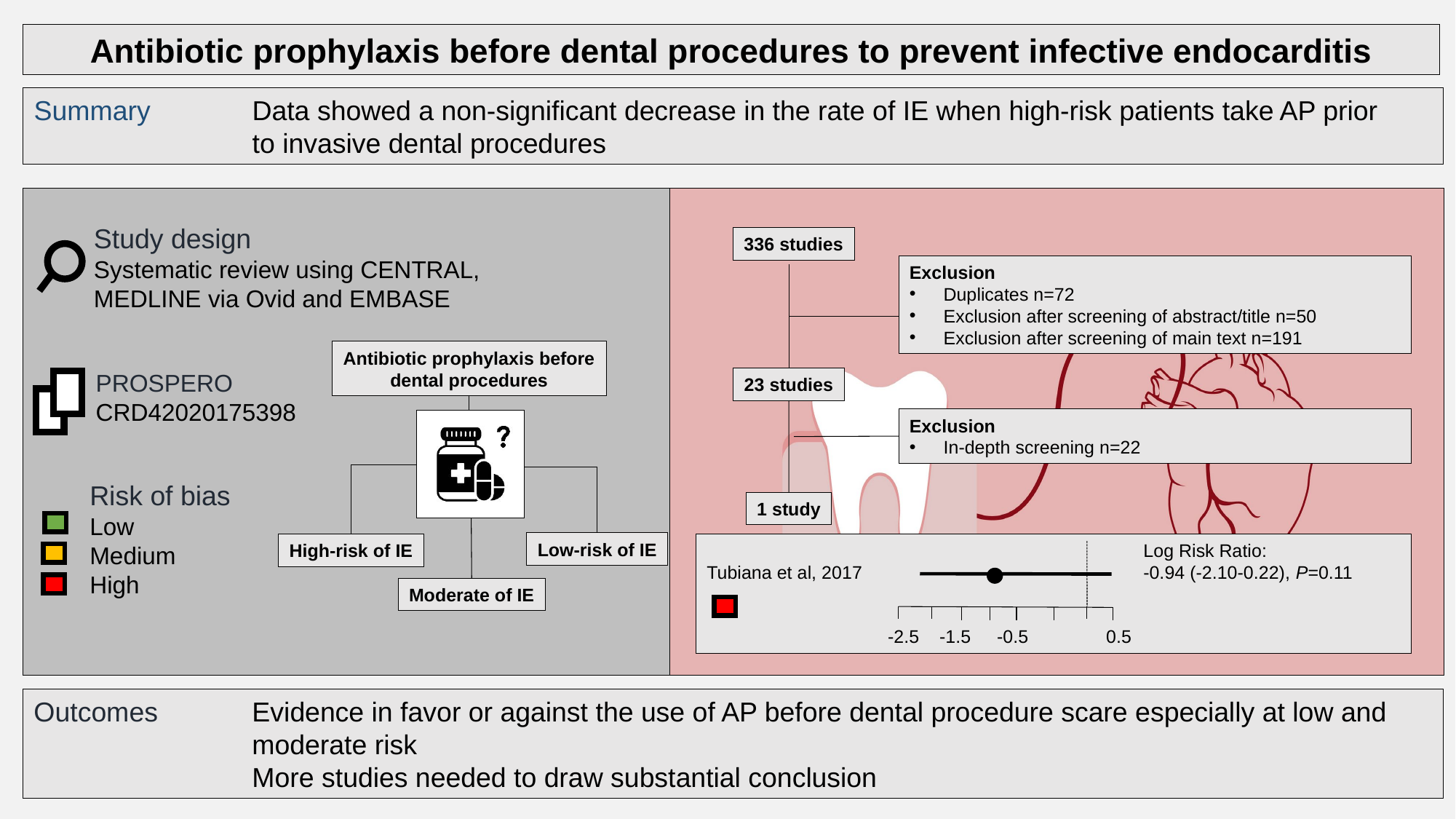

Antibiotic prophylaxis before dental procedures to prevent infective endocarditis
Summary 	Data showed a non-significant decrease in the rate of IE when high-risk patients take AP prior 		to invasive dental procedures
Study design
Systematic review using CENTRAL, MEDLINE via Ovid and EMBASE
336 studies
Exclusion
Duplicates n=72
Exclusion after screening of abstract/title n=50
Exclusion after screening of main text n=191
Antibiotic prophylaxis before
dental procedures
PROSPERO
CRD42020175398
23 studies
Exclusion
In-depth screening n=22
Risk of bias
Low
Medium
High
1 study
Low-risk of IE
				Log Risk Ratio:
Tubiana et al, 2017			-0.94 (-2.10-0.22), P=0.11
High-risk of IE
Moderate of IE
-2.5 -1.5	-0.5 	0.5
Outcomes 	Evidence in favor or against the use of AP before dental procedure scare especially at low and 		moderate risk
		More studies needed to draw substantial conclusion
